# Knowledge, attitude, practice in hypertension management and their association with blood pressure control: A cross-sectional study

**DOI:** 10.6026/973206300222328

**Published:** 2026-04-30

**Authors:** Harshitha Kumar, Midha Mehmood Shaikh, Rajesh Dehankar, Aisha Mohammed Kutty

**Affiliations:** 1 Department of OHC, U R Lifecare Patholab & Clinic, Lingarajapuram, Bangalore, Karnataka, India; 2 Department of Obstetrics and Gynaecology, Bharati Vidyapeeth medical college, Pune, India; 3 Department of Anatomy, Professor, NKP Salve Institute of Medical Sciences & RC and Lata Mangeshkar Hospital, Nagpur, India; 4 Department of Oncology and Haematology, Portsmouth University Hospital NHS trust, United Kingdom

**Keywords:** Hypertension, patient knowledge, blood pressure monitoring, adherence, lifestyle modification

## Abstract

Inadequate patient understanding of hypertension remains a major barrier to optimal blood pressure control. Therefore, it is of interest to evaluate knowledge, attitude and practice (KAP) related to hypertension management among 150 adults receiving antihypertensive therapy and examined its association with objectively measured blood pressure. A validated 25-item KAP questionnaire was administered and blood pressure was measured using a standardized protocol. Only 38% demonstrated good knowledge, 46% showed positive attitudes and 43% reported appropriate practices. Mean systolic and diastolic blood pressures were 142.3 ± 15.1 mmHg and 89.5 ± 10.3 mmHg, respectively. Total KAP scores showed significant inverse correlations with systolic (r = -0.39) and diastolic (r = -0.34) blood pressure (p < 0.05). Thus, we show that better patient understanding is associated with improved blood pressure control and support the need for structured education and monitoring strategies.

## Background:

Hypertension is a major global public health concern and a leading risk factor for cardiovascular, renal and cerebrovascular disease [1]. More than one billion adults worldwide are affected and control rates remain suboptimal. Poor awareness, delayed diagnosis and inadequate long-term management contribute to persistent uncontrolled blood pressure [2]. Effective hypertension management requires sustained medication adherence and lifestyle modification. Dietary salt restriction, physical activity, weight control and stress reduction are essential components of therapy [3]. However, adherence to these recommendations remains inconsistent. Limited patient knowledge and misconceptions about chronic treatment frequently undermine long-term control [4]. Knowledge, Attitude and Practice (KAP) frameworks are widely used to evaluate patient understanding and behavioural patterns [5]. Higher knowledge levels have been associated with improved medication adherence and better blood pressure outcomes. Conversely, inadequate understanding contributes to therapeutic non-adherence and increased cardiovascular risk [6]. Home blood pressure monitoring provides objective reinforcement of behavioural change and enhances patient engagement [7]. Regular monitoring improves self-efficacy and promotes adherence to treatment. Integration of patient education with monitoring strategies has demonstrated improved control rates in multiple settings [8]. Therefore, it is of interest to evaluate patient comprehension of hypertension management using KAP assessment and correlate these findings with objectively measured blood pressure.

## Materials and Methods:

This cross-sectional study took place in the outpatient department of a tertiary care hospital. The duration of the study was six months, from March 2025 to August 2025. A total of 150 adult patients with a diagnosis of hypertension who had been receiving antihypertensive medications for at least six months were included in the study, following informed consent. Patients with secondary hypertension, chronic kidney disease stage 4 or higher, pregnancy, or impaired cognition were excluded. The following demographic data were collected: age, gender, duration of hypertension, comorbidities and medication use. The patients' understanding of hypertension was assessed through a previously validated Knowledge, Attitude and Practice (KAP) questionnaire composed of 25 questions. The KAP questionnaire assess patients' knowledge of hypertension (10 questions); patient attitudes towards the management of the disease (8 questions); and the patient's lifestyle and medication (7 questions). In the KAP questionnaire, each correct answer got a score of 1 and each incorrect answer got a score of 0. Then we categorized patient's total scores as poor, average and good. Blood pressure was measured using a standard sphygmomanometer according to guidelines of the American Heart Association. At each visit, two readings separated by five minutes were obtained in seated and resting patients. Home blood pressure monitoring values, if available, were recorded in order to supplement the office values. The data was analyzed with SPSS (version 26.0). Descriptive statistics are presented as mean ± SD for continuous variables and as percentages for categorical variables. The relationship between KAP scores and average systolic and diastolic blood pressure was tested by Pearson correlation. The p-value < 0.05 was defined to be statistically significant. Subgroup analysis conducted was age groups, gender and duration of hypertension. The study adhered to the Declaration of Helsinki. Ethical approval obtained from the institutional ethics committee (IEC No: 2025/HTN/012). The sample size of 150 was calculated based on prior studies showing a moderately correlated moderate correlation (r = 0.3) between patient knowledge and blood control. Power was set at 80% and α = 0.05.

## Results:

Among the 150 participants, 82 were male and 68 were female. Mean age of participants was 55.8 ± 10.2 years. The mean duration of their hypertension was 7.9 ± 4.1 years. Participants' mean systolic blood pressure (BP) was 142.3 ± 15.1 mmHg and mean diastolic blood pressure was 89.5 ± 10.3 mmHg. Mean Knowledge, Attitude and Practice (KAP) score for the participants was 16.4 ± 4.2 indicating modest awareness. Good knowledge (38% of participants), positive attitude (46% of participants) and appropriate practices (43% of participants) were documented. Total KAP scores were inversely related to the means systolic (r = -0.39) and diastolic (r = -0.34) BP (p <0.05), which indicates that a better understanding of hypertension was related to better control of BP. Demographic and clinical characteristics are summarized in Table 1 (see PDF) for the 150 participants included in the study. The sample consisted of 82 males (55%) and 68 females (45%), with a mean age of 55.8 (SD ± 10.2) years. The mean history of hypertension was 7.9 (SD ± 4.1) years and the mean systolic blood pressure during the assessment was 142.3 (SD ± 15.1) mmHg while mean diastolic blood pressure was 89.5 (SD ± 10.3) mmHg, demonstrating moderate-to-poor blood pressure control during the data collection time. Table 2 (see PDF) summarizes the participants' hypertension management knowledge which indicated 38% had good knowledge, 42% had average knowledge and 20% had poor knowledge. While the literature suggests that a considerable proportion of patients possess moderate knowledge about their hypertension condition, the data from this study also suggests that many well-informed patients state having poor knowledge about their hypertension condition, which impacts the management of the disorder. Table 3 (see PDF) illustrates the prevalence of positive attitudes to hypertension management among participants indicating that 46% had positive attitudes towards their hypertension management, while 34% replied neutral attitudes and 20% had negative attitudes about their management. These findings illustrated that less than half of participants demonstrated positive attitudes about adherence to treatment or lifestyle changes to managing their hypertension. Table 4 (see PDF) shows respondents' self-reported engagement in behaviour's associated with the management of their hypertension. 60% were adherent to medication, 48% reported engaging in a dietary change and, 45% reported regular physical activity. This point to a discrepancy between respondents' behaviors related to lifestyle and adherence to prescribed medication and an awareness of the importance of these behaviors and therefore highlights the need for behavioral interventions. Table 5 (see PDF) represents knowledge level and systolic BP data, showing that people with a high knowledge level reported a mean systolic BP of 136.5 ± 12.2 mmHg versus 143.7 ± 14.5 mmHg average knowledge and 150.2 ± 15.1 mmHg poor knowledge. In other words, the more knowledgeable they were about their hypertension, the better the systolic BP control among subjects. Table 6 (see PDF) indicates the relationship between knowledge level and diastolic BP, showing the mean diastolic BP of those with good knowledge to be 85.4 ± 8.9 mmHg against average knowledge of 90.1 ± 10.2 mmHg and poor knowledge of 94.3 ± 11.0 mmHg. These data continue to support the link between patient knowledge and optimal BP control. Table 7 (see PDF) depicts the relationship between total KAP scores and BP and includes the statistical correlation, including a significant inverse correlation between total KAP score and BP (systolic r = -0.39, diastolic r = -0.34), p < 0.05. These data imply that the improved patient knowledge, attitude and practices led to improved BP levels and ultimately improved hypertension control.

## Discussion:

This study evaluated patient knowledge, attitudes and practices related to hypertension management and examined their association with measured blood pressure. The findings demonstrate moderate overall awareness but substantial gaps in optimal behavioural practices. Only 38% of participant's demonstrated good knowledge and fewer than half reported consistent lifestyle modification. The mean systolic and diastolic blood pressures indicate suboptimal control within this cohort. Despite being on treatment for an average of nearly eight years, many participants did not achieve target levels [9]. This highlights the persistent gap between pharmacological prescription and effective long-term disease control [10]. The inverse correlation between KAP scores and both systolic and diastolic blood pressure is clinically significant. Participants with better knowledge demonstrated lower mean blood pressure values [11]. This supports evidence suggesting that informed patients are more likely to adhere to medication and lifestyle recommendations. Improved understanding enhances self-efficacy and engagement in chronic disease management [12]. Medication adherence was reported by 60% of participants. This suggests that adherence remains incomplete despite prolonged disease duration. Lifestyle adherence was even lower, particularly for dietary modification and physical activity. Behavioural change requires continuous reinforcement and structured support rather than one-time counselling [13]. The association between knowledge and improved blood pressure underscores the importance of patient-centred education. Structured counselling, clear communication and repeated reinforcement are essential. Educational strategies must address misconceptions, emphasize long-term risk and provide practical behavioural guidance [14]. Home blood pressure monitoring may further strengthen adherence. Objective feedback reinforces behavioural change and increases patient accountability. Integration of monitoring with education may yield sustained improvements in control [15]. The study contributes contemporary data by correlating structured KAP assessment with measured blood pressure in a real-world outpatient population. It highlights that knowledge deficits remain prevalent despite ongoing treatment. Importantly, the findings quantify the relationship between comprehension and physiological outcomes rather than relying solely on self-reported adherence [16]. Several limitations should be acknowledged. The cross-sectional design precludes causal inference. Self-reported practices may be influenced by recall bias. However, objective blood pressure measurements strengthen the validity of the associations observed. Overall, the findings emphasize that hypertension management requires more than pharmacological treatment. Educational reinforcement, behavioural counselling and monitoring strategies must be integrated into routine care. Addressing knowledge deficits may significantly improve long-term cardiovascular risk reduction.

## Conclusion:

Patient knowledge, attitudes and practices significantly influence blood pressure control. Higher KAP scores are associated with lower systolic and diastolic blood pressure. Structured patient education, regular reinforcement and home monitoring should be integrated into routine hypertension management to improve long-term outcomes.
